# Effects of sportsmetrics training on biomechanical and functional performance outcomes related to ACL injury risk: a systematic review and meta-analysis

**DOI:** 10.1186/s13102-026-01948-7

**Published:** 2026-08-08

**Authors:** Mostafa Jalili Bafrouei, Fatemeh Zahra Maleki Gohar, Seyed Hamed Mousavi, Mohamad Rostami, Florian Forelli

**Affiliations:** 1 Department of Sport Injuries and Biomechanics, Faculty of Sport Sciences and Health, University of Tehran, Tehran, Iran; 2 Monash Exercise Neuroplasticity Research Unit, Department of Physiotherapy, School of Primary and Allied Care, Faculty of Medicine, Nursing and Health Science, Monash University, Melbourne, Australia; 3 Haute-Ecole Arc Santé, Hes-So University of Applied Sciences and Arts Western Switzerland, Delémont, 2800 Switzerland; 4 Orthopaedic Surgery Department, Clinic of Domont, Ramsay Healthcare, @Ortholab, Domont, France; 5 Société Française Des Masseurs-Kinésithérapeutes Du Sport Lab, Pierrefitte-Sur-Seine, France

**Keywords:** Athletic performance, Dynamic knee valgus, Landing biomechanics, Neuromuscular training

## Abstract

**Background:**

This meta-analysis aimed to evaluate the effects of SportsMetrics training (SMT) on ACL injury-related biomechanical deficits and functional performance outcomes in athletes.

**Methods:**

Literature searches were conducted in four databases from inception to May 10, 2026. The RoB 2 and ROBINS-I tools were used to assess the risk of bias, while meta-analyses and sensitivity analyses were performed using CMA 4 software. The GRADE approach was applied to evaluate the quality of evidence. Primary outcomes were dynamic knee valgus (DKV) and the Landing Error Scoring System (LESS). Secondary outcomes included core strength, agility, dynamic balance, speed, and power.

**Results:**

Nine studies (310 athletes, predominantly female) were included. SMT was associated with changes in surrogate biomechanical outcomes, including DKV (SMD: -1.09, *p* < 0.0001) and LESS (SMD: -1.08, *p* < 0.0001), as well as functional performance outcomes: agility, dynamic balance, power, and speed. Core strength (SMD: 0.57, *p* = 0.154) showed non-significant changes.

**Conclusions:**

SMT was associated with improvements in several biomechanical and functional outcomes. Higher-quality RCTs and prospective injury registration outcomes are required to confirm these findings. However, the relationship between improvements in these surrogate outcomes and actual ACL injury reduction remains unestablished.

**Trial registration:**

This study was a priori registered at PROSPERO (CRD420261389110).

**Supplementary Information:**

The online version contains supplementary material available at https://doi.org/10.1186/s13102-026-01948-7.

## Key Messages

• SMT is associated with improvements in several biomechanical and functional performance outcomes in athletes.

• Evidence is based on low to very low certainty studies with predominance of quasi-experimental designs and no prospective injury-registration data.

• Current findings support short term movement and performance changes but should not be interpreted as evidence of ACL injury risk reduction

**Supplementary Information:**

The online version contains supplementary material available at https://doi.org/10.1186/s13102-026-01948-7.

## Introduction

Sports such as soccer, basketball, volleyball, handball, and futsal involve frequent jumping, landing, cutting, and rapid changes of direction, exposing athletes to a high risk of lower-extremity injuries [1]. Epidemiological studies report that 60–90% of injuries in soccer and 26–60% in volleyball occur in the lower extremity, with the knee being the most commonly injured joint [2–4]. Female athletes demonstrate nearly 3.5 times higher knee injury rates than males, while more than 60% of ACL injuries occur through non-contact mechanisms [5]. Biomechanical deficits such as excessive dynamic knee valgus (DKV), reduced hip and knee flexion, poor trunk control, and neuromuscular impairments during landing have been associated with movement patterns that may contribute to increased knee loading and ACL injury risk during high-risk tasks [6, 7]. Beyond the time loss associated with sports, ACL injuries are linked to an increased risk of recurrent injury, reduced athletic performance, long-term functional limitations, and early onset of knee osteoarthritis [8].

Consequently, identifying modifiable biomechanical factors associated with ACL injury risk has become a major focus in sports injury prevention research [9]. Among these factors, dynamic knee valgus (DKV) is commonly used as a biomechanical movement variable, while the Landing Error Scoring System (LESS) is a clinical screening tool used to assess movement quality during landing tasks. Both are frequently used as surrogate measures of movement patterns associated with lower-extremity injury risk [6, 10]. Excessive DKV reflects impaired neuromuscular control and abnormal frontal-plane knee loading [11]. Similarly, higher LESS scores indicate deficient landing mechanics characterized by increased knee valgus, poor trunk positioning, and inadequate shock absorption strategies [12]. In addition, functional performance variables such as agility [13], dynamic balance [14], speed, and power are essential not only for athletic success but also for maintaining efficient and safe movement patterns during sport-specific activities [15]. Deficits in these variables may compromise neuromuscular control and further elevate injury susceptibility.

To address these deficits, neuromuscular injury-prevention programs have been developed, among which SportsMetrics training (SMT) has emerged as one of the most comprehensive and sport-specific approaches [16, 17]. Several other ACL injury-prevention programs, such as FIFA 11+ [18], Prevent Injury and Enhance Performance (PEP) [19], and Knee Injury Prevention Program (KIPP) [20], have also shown beneficial effects; however, these programs differ in their exercise components, implementation strategies, and primary objectives. The present review focused specifically on SMT because it represents a standardized neuromuscular training approach emphasizing plyometric exercises, jump-landing retraining, strength, agility, and movement-control training. Importantly, this review aimed to evaluate the effects of SMT on biomechanical and functional performance outcomes rather than to compare different ACL injury-prevention programs.

SMT typically incorporates progressive plyometric loading, jump-landing retraining, multidirectional movement tasks, strength exercises, agility drills, and sport-specific neuromuscular training [21, 22], whereas conventional prevention protocols primarily emphasize isolated strengthening or brief warm-up exercises [23]. In the included studies, these components were associated with improvements in biomechanical and performance-related outcomes; however, the underlying mechanisms are interpreted based on theoretical neuromuscular and biomechanical models rather than directly measured causal pathways. SMT may be associated with improvements in functional performance and movement quality in athletes, potentially reflecting adaptations that are commonly interpreted as consistent with enhanced neuromuscular control, although neuromuscular control itself was not directly measured in the included studies.

To the authors’ knowledge, this is the first systematic review and meta-analysis to quantitatively synthesize the effects of SMT on ACL injury-related biomechanical outcomes and functional performance variables in athletes. Therefore, the present study aimed to systematically evaluate the effects of SMT on primary biomechanical outcomes, including DKV and LESS, as well as functional performance variables such as core strength, agility, dynamic balance, speed, and power in athletes. It was hypothesized that SMT may be associated with improvements in lower-extremity alignment and landing mechanics, as well as sport-specific functional performance outcomes in athletes. However, these changes should be interpreted as modifications in biomechanical and performance-related variables, and not as direct evidence of reduced ACL injury incidence.

## Method

### Study design

This systematic review and meta-analysis was conducted in accordance with the Preferred Reporting Items for Systematic Reviews and Meta-Analyses (PRISMA) 2020 guidelines [24]. The objective was to identify, critically appraise, and synthesize RCTs and quasi-experimental studies with a control group comparing SMT between healthy player participants. The review protocol was prospectively registered in PROSPERO (CRD420261389110).

### Search strategy

Relevant studies were identified through a comprehensive search of four electronic databases: PubMed, Web of Science, Scopus, and Embase. The search was conducted on 10 May 2026. To ensure comprehensiveness, both Sportsmetrics-specific terms and broader neuromuscular training terminology were included in the search strategy, as these interventions often share common components and are inconsistently labeled across the literature. Study inclusion was subsequently restricted to interventions that clearly aligned with structured Sportsmetrics protocols or equivalent neuromuscular training programs incorporating core SMT elements such as plyometric jump-landing drills, strength, agility, and movement control exercises.

The search strategy was based on broad terms and related synonyms organized: #1 Athlete* OR player* OR “soccer* play*” OR sportperson* OR “football* play*” OR “volleyball* play*” OR “futsal* play*” OR “basketball* play*”, #2 “Sports-metric train*” OR “Sports-metric exercis*” OR “Sportsmetrics Program*” OR Sportsmetric* OR “neuromuscular train*” OR “neuromuscular exercis*” OR NMT OR NME OR “NM exercis*” OR “NM train*” OR NMP OR “NM program” #3 Landing* OR hop OR performanc* OR jump* OR balance* OR agility* OR function* OR pain OR activit* OR power* OR gait* OR speed* OR “core stabili*” OR strength* OR “landing error screen system” OR LESS OR kinematic* OR “dynamic knee valgus” OR DKV OR “knee abduction” OR “knee abd*” OR “knee displacement” OR “knee biomechanic*” OR biomechanic*), #4 (1 AND 2 AND 3). Additionally, reference lists from previous systematic reviews on Sport-metric and neuromuscular training in athletic populations were manually searched to ensure the inclusion of all relevant studies.

### Eligibility criteria

Studies were included according to the following criteria (PICO):

- Population: Athletes (soccer, volleyball, futsal, basketball) aged ≥ 12 years, with or without DKV, but free from acute lower-extremity injury at enrollment.
- Intervention: SMT is defined as a structured program combining plyometrics, jump-landing retraining, agility, and strengthening, delivered for at least 6 weeks.
- Comparator: Active control (usual or regular sport training).
- Outcomes: The primary outcome was dynamic knee valgus (DKV), assessed using a double-leg squat and two-dimensional (2D) video-based analysis, and the Landing Error Scoring System (LESS), evaluated using a video-based drop-jump test. While both methods are widely used in clinical and field-based settings due to their feasibility, it should be acknowledged that 2D assessments of DKV have limitations in biomechanical precision and ecological validity compared with three-dimensional motion analysis. In addition, although LESS is a standardized and validated clinical screening tool, there was some variation in implementation and reporting across the included studies, and not all studies provided detailed information regarding adherence to identical scoring protocols.
- Secondary outcomes included core strength (McGill’s test and core strength assessments), agility (T-test and Illinois agility test), dynamic balance (Y-Balance Test), lower-limb power (vertical jump tests and Sargent jump test), and speed (dribbling tests, Moore–Christian shooting test, and sprint tests over 20-, 40-, and 50-yard distances). Although different tests were used across studies, power-related outcomes were considered proxy measures of lower-extremity explosive performance and were grouped under a common construct for synthesis purposes.
- Study design: Randomized controlled trials and quasi-experimental studies with a control group.
- Language and publication status: Peer-reviewed articles published in all languages.

Studies involving interventional rehabilitation programs, animal models, conference abstracts, editorials, reviews, or case reports were excluded.

All records retrieved from the database searches were imported into EndNote (version 21), and duplicates were removed. Following study selection, the included studies were imported into the Comprehensive Meta-Analysis software (CMA, version 4) for data analysis. MJB and FZMG independently screened the titles and abstracts to identify relevant studies. A calibration exercise was conducted on a random subset of studies before full screening to ensure consistency. Inter-rater reliability during both title/abstract screening and full-text review was quantified using Cohen’s kappa (κ), with κ ≥ 0.80 interpreted as strong agreement. Disagreements were resolved by consensus or through consultation with the reviewer, SHM. Any missing or incomplete data in the included studies were addressed by contacting the corresponding authors when possible. If sufficient data were not provided, the conference study was excluded from the analysis.

### Data extraction

MJB and FZMG independently collected information from the included studies using a standardized extraction form to ensure consistency. Agreement between reviewers was quantified using Cohen’s kappa (κ). An a priori threshold of κ ≥ 0.75 was considered acceptable reliability. The observed inter-rater agreement was κ = 0.79, indicating substantial agreement between reviewers. SHM resolved discrepancies. The following information was recorded: author and year, study design, participants’ demographics (age, sex, height, weight), SMT protocol, tools, intervention and duration, inclusion and exclusion criteria, funding information, and outcome measures, including DKV, LESS, core strength, agility, dynamic balance, power, or speed.

### Methodological quality assessment

Methodological quality assessment was considered a complementary evaluation of the overall methodological characteristics and reporting features of the included studies. Because methodological quality and risk of bias address different aspects of study evaluation, risk of bias was assessed separately using design-specific tools. RCTs were evaluated using the PEDro scale [25]. PEDro scores were interpreted as poor (< 4), fair (4–5), good (6–8), or excellent (9–10) [26]. Methodological quality assessment was conducted independently by MJB and MR. Inter-rater agreement was assessed using Cohen’s kappa coefficient and interpreted according to Landis and Koch’s criteria (κ < 0.20 = poor; 0.21–0.40 = fair; 0.41–0.60 = moderate; 0.61–0.80 = substantial; > 0.80 = almost perfect agreement).

The methodological quality of the included non-randomized studies was assessed using the appropriate Joanna Briggs Institute (JBI) Critical Appraisal Checklists [27]. The JBI tool evaluates methodological domains including participant selection, measurement validity and reliability, management of confounding factors, and appropriateness of statistical analyses. Each item was rated as “yes,” “no,” “unclear,” or “not applicable,” and no overall numerical score was calculated in accordance with JBI recommendations. Disagreements were resolved through open discussion and a consensus-based approach. Methodological quality assessments were considered during the interpretation of pooled and narrative findings to aid the evaluation of the consistency and strength of the evidence. However, methodological quality did not influence the statistical weighting of studies in the meta-analysis.

### Risk of bias

Risk of bias assessment was performed separately from methodological quality assessment because these approaches evaluate distinct methodological domains. While methodological quality assessment focuses on predefined methodological characteristics and reporting features of the included studies, risk-of-bias assessment evaluates potential sources of systematic bias that may influence the validity and interpretation of study findings. Accordingly, RoB 2 was used for RCTs, and ROBINS-I was applied for non-randomized studies.

Risk of bias was independently assessed by two reviewers (MJB and MR) using the appropriate Cochrane risk-of-bias tools according to study design. RCTs were evaluated using the RoB 2, which assesses bias across five domains [28], including the randomization process, deviations from intended interventions, missing outcome data, outcome measurement, and selection of the reported result. Each domain was judged as “low risk of bias,” “some concerns,” or “high risk of bias.”

Quasi-experimental studies were assessed using ROBINS-I [21], which evaluates bias across seven domains, including confounding, participant selection, intervention classification, deviations from intended interventions, missing data, outcome measurement, and selective reporting. Overall judgments were categorized as “low,” “moderate,” “serious,” or “critical” risk of bias. Any disagreements between reviewers were resolved through discussion and consensus.

### Publication bias and certainty of evidence

Publication bias was assessed using visual inspection of funnel plots, Begg’s test, Egger’s regression test, and trim-and-fill analysis. For meta-analyses including fewer than 10 studies, Begg’s test, Egger’s regression test, and trim-and-fill methods were applied [29]. Due to the limited number of included studies, the results of publication bias assessments were interpreted with caution because of low statistical power.

The certainty of evidence was evaluated using the GRADE approach. This framework assesses evidence across five domains: risk of bias, inconsistency, indirectness, imprecision, and publication bias [30]. Two reviewers independently conducted the GRADE assessment, and disagreements were resolved through discussion or consultation with a third reviewer.

### Data collection

MJB and FZMG extracted all data from the included studies, with SHM verifying the accuracy of the extracted data. The number of sessions, study design, variables, number of participants, and their characteristics (age, sex, height, and mass), as well as the tools used, were systematically extracted from the included studies.

### Synthesis of results

Meta-analyses were conducted using standardized mean differences (SMD; Hedges’ g) with 95% confidence intervals (CI), calculated using a random-effects model in CMA (version 4). A random-effects approach was chosen due to expected heterogeneity in study populations, cognitive-motor performance, and outcome measures. Meta-analysis was performed when at least two studies reported comparable outcomes using similar methodologies. Statistical heterogeneity was assessed using the I² statistic, with values of 25%, 50%, and 75% representing low, moderate, and high heterogeneity, respectively. Pre-planned subgroup analyses were considered for sport type (soccer vs. volleyball, basketball), baseline DKV status (present vs. absent), and study design (RCTs vs. quasi-experimental). However, due to the small number of studies and inconsistent reporting, only descriptive subgroup comparisons were performed in the discussion. Given the inclusion of both RCT and quasi-experimental studies, sensitivity analyses were performed to evaluate the stability and robustness of the pooled results by examining the impact of each study on the overall effect estimates.

To minimize the risk of duplicate participant inclusion, studies from the same research groups were carefully evaluated for potential overlap in participant samples, intervention periods, and study characteristics. To address the potential overlap among studies originating from similar research groups, we carefully examined the available information regarding recruitment periods, institutions, sport types, and participant characteristics. Four studies [31–34] originated from the same research group (Mohammadi et al.) but enrolled distinct participant cohorts based on different sport types (volleyball, soccer, and basketball) and recruitment periods (2020, 2021, and 2022). Additionally, two studies (Saki et al.) [35, 36] were conducted at different time points (2019 and 2020) and included different participant samples. Details of the overlap assessment are provided in Supplementary File 1. Based on the available information, no evidence of participant overlap was identified across any of the included studies.

## Results

### Study selection

**Fig. 1:**
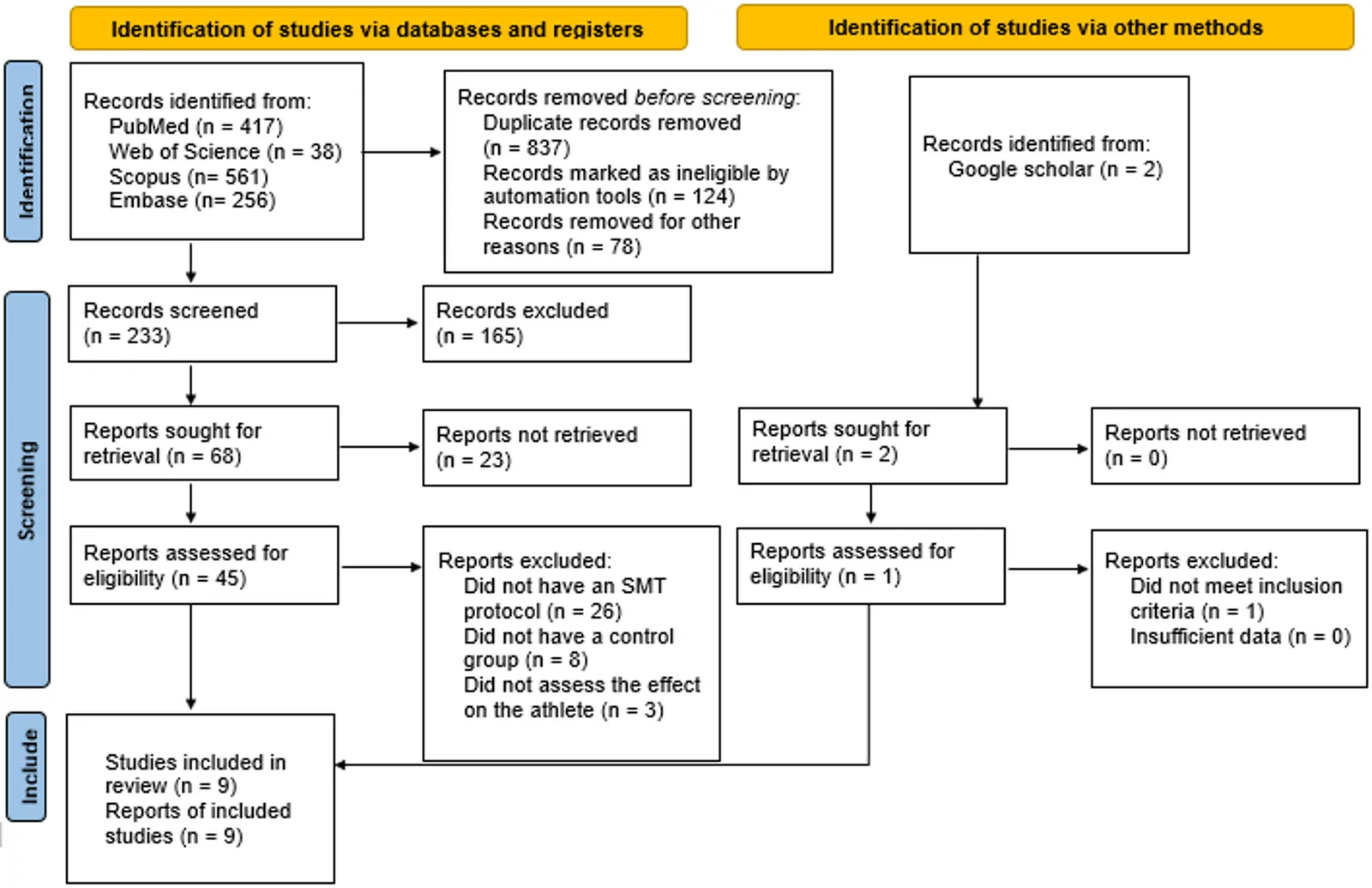
Flow diagram of the included studies

The main literature search yielded a total of 1272 items (PubMed (417 studies), Web of Science (38), Scopus (561), and Embase (256)) from which 435 items remained after duplicate removal. After screening the titles and abstracts, 9 studies were included. Figure 1 illustrates the flow diagram of the selection process, showing the number of excluded studies at each stage. Any missing or incomplete data in the included studies were addressed by contacting the corresponding authors when possible. If sufficient data were not provided, the study was excluded from the analysis. The primary exclusion criteria comprised: absence of established SMT (*n* = 26), lack of control group implementation (*n* = 8), and failure to evaluate athlete outcomes (*n* = 3).

### Study characteristics

Table 1 presents the characteristics of the included studies. The publication years ranged from 2015 to 2025 (median: 2020), with 88.88% (8/9) of the studies published after 2020. Collectively, the nine included studies comprised a total sample size of 310 participants. This relatively small cumulative sample size should be considered when interpreting the findings, as limited sample sizes may reduce statistical power, increase the uncertainty of pooled effect estimates, and potentially lead to inflated effect sizes. Consequently, the precision and robustness of the meta-analytic results may be affected, and the findings should be interpreted with appropriate caution.

**Table 1:** Characteristics of the included studies

Authors	Study design	Participation	Intervention	Duration	Sample size and sex	Outcomes	Tools
Kalantariyan et al. 2025 [37]	Quasi-experimental	Futsal players	Experimental group (EG): Sport-metrics training, control group (CG): regular futsal training	6 Weeks, 3 times per week	EG: 15 males, CG: 15 males	Static balance, dynamic balance, agility, power, dribbling speed, and shooting	Illinois Agility Test, Stork Test, Y-Balance Test, Triple Hop Test, Dribbling Test, Moore-Christian Shooting Test
Mohammadi et al. 2024 a [33]	Quasi-experimental	Volleyball players with a dynamic knee valgus (DKV)	EG: Sport-metrics training, CG: normal exercises	6 Weeks, 3 times per week	EG: 15 females, CG: 15 females	Landing error during Landing Error Scoring System (LESS), dynamic knee valgus (DKV) during Double Leg Squat (DLS)	DLS test, LESS
Mohammadi et al. 2024 b [38]	Quasi-experimental	Soccer players with DKV	EG: Sport-metrics training, CG: normal training	6 Weeks, 3 times per week	EG: 12 females, CG: 12 females	Core strength, postural stability, DKV during DLS	Core strength test (trunk flexor, trunk extensor, right side bridge, left side bridge), Y balance, LESS
Mohammadi et al. 2023 [34]	Quasi-experimental	Basketball with DKV	EG: Sport-metrics training, CG: regular training	6 Weeks, 3 times per week	EG:15 females, CG: 15 females	Core strength and stability, balance, agility, speed, and power	McGill’s Y Balance, T, and Illinois agility, Speed tests 20-Yard and 40-Yard, Vertical jump tests
Saki et al. 2022 [36]	RCT (Randomized Clinical Trial)	Soccer players	EG: Sport-metrics training, CG: normal exercises	6 Weeks, 3 times per week	EG: 12 males, CG: 12 males	Core stability, lower limb function	McGill test, Tuck jump, Hop
Mohammadi et al. 2022 [31]	Quasi-experimental	Volleyball players with DKV	EG: Sport-metrics training, CG: normal exercises	6 Weeks, 3 times per week	EG: 12 females, CG: 12 females	Landing error during LESS, DKV during DLS	DLS, LESS
Shahrjerdi et al. 2021 [32]	Quasi-experimental	Volleyball players	EG: Sport-metrics training, CG: regular volleyball exercises	6 Weeks, 3 times per week	EG: 13 females, CG: 12 females	Landing technique	Landing error during LESS
Saki et al. 2021 [35]	RCT	Young soccer players	EG: Sport-metrics training, CG: normal training	6 Weeks, 3 times per week	EG: 14 males, CG: 15 males	Speed, agility, and power tests	50-yard sprint, Illinois, and sergeant tests
Singh et al. 2015 [39]	Quasi-experimental	Elite basketball players	EG: Sport-metrics training, CG: normal exercises	6 Weeks, 3 times per week	EG: 20 females, CG: 20 females	Lower limb alignment on a drop-jump test, performance, and agility	Video drop-jump test, Vertical jump test, Illinois agility test

Authors	Age (year)	Weight (kg)	Height (cm)	BMI (kg/m2)	Fund	Intervention Protocols
Kalantariyan et al. 2025 [37]	EG: 11.1 ± 1.4, CG: 10.8 ± 1.3	EG: 52.7 ± 4.6, CG: 54.4 ± 5.8	EG: 154.5 ± 6.2, CG: 156.2 ± 11.3	N/R	N/A	A 6-week Sport-metric program (18 sessions; 3 sessions/week) was structured into Agility & Reaction, Endurance/Speed/Resistance, Ladder, Plyometrics, and Strength. Agility & Reaction progresses from Zig-zag/obstacle runs (W1-2) to Shuttles and speed/stop drills (W3-4), and then to Nebraska/Illinois/T-drills with rotational reactive running (W5-6). Ladder drills are performed throughout the program (2 reps per drill), while Plyometrics follow a fixed prescription (3 sets × 5 reps). Strength increases across the weeks: 30 s (W1-2), 45 s (W3-4), 60 s (W5-6). Overall, conditioning (Endurance/Speed/Resistance) gradually shifts toward more advanced speed, resistance, and cutting/turning shuttle patterns.
Mohammadi et al. 2024 a [33]	EG: 20.4 ± 1.9, CG: 21.3 ± 2.5	EG: 57.46 ± 6.73, CG: 60.8 ± 7.89	EG: 166.46 ± 6.34, CG: 169.86 ± 6.35	EG: 20.69 ± 1.62, CG: 20.98 ± 1.46	N/A	The Sports-metrics training protocol for female volleyball players is a 6-week program combining plyometric and agility drills with progressive intensity. Each week includes several sessions featuring sport-specific movements such as resisted partner pushes, fast and backward running, ladder and rotation drills, shuttle and reaction-based drills (e.g., boxing to coach commands), and jumping/footwork exercises. In parallel, a strength component is performed across the sessions using resistance-band and partner-resisted exercises (e.g., squats, lunges, single-leg calf raises, hamstring bridges, scapular work, core stability, hip flexor/abductor and boxing-footwork). Time per strength exercise progresses from 30 s in sessions 1–6 to 45 s in sessions 7–12, and then to 60 s in sessions 13–18.
Mohammadi et al. 2024 b [38]	EG: 20.1 ± 2.0, CG: 19.7 ± 1.7	EG: 56.2 ± 4.9, CG: 53.5 ± 5.3	EG: 164.1 ± 7.3, CG: 162.3 ± 5.3	EG: 20.8 ± 1.9, CG: 20.3 ± 1.8	N/A	The Sports-metrics training program follows a 6-week progression with three sessions per week, moving from basic hip/knee control to landing skill and then to more explosive, dynamic stability. In Phase 1 (weeks 1–2), the emphasis is on controlled positions and light plyometrics using exercises such as half-squats, static lunges, plus hamstring and glute stretching and related core stability work. In Phase 2 (weeks 3–4), the program increases plyometric demand and landing control through drills like Drop Jump, Jump & Reach, Box Jump, and Side Hop. In Phase 3 (weeks 5–6), training becomes more advanced and power-oriented with Bounding and cutting drills, combined with core/balance strengthening such as planks, bridges, and Bosu ball drills, to improve dynamic control and reduce knee valgus during movement.
Mohammadi et al. 2023 [34]	EG: 15.40 ± 1.24, CG: 16.06 ± 1.27	N/R	EG: 161.67 ± 0.59, CG:162.60 ± 0.41	EG: 20.82 ± 1.23, CG: 21.39 ± 0.98	N/A	The program is a progressive neuromuscular training plan structured in three phases over six weeks. In phase one (Weeks 1–2), the focus is on static balance and fundamental strength through exercises such as single-leg stance, basic squats, lunges, and simple core-st stabilization. During phase two (Weeks 3–4), the program introduces dynamic and multi-directional stability work, including forward and lateral jumps, balance-board tasks, and more advanced strength movements to enhance control during motion. In phase three (Weeks 5–6), emphasis shifts to plyometric and sport-specific agility exercises involving rapid direction changes, jumping, and dual-task drills to further improve neuromuscular coordination and reduce injury risk. Overall, intensity and complexity gradually increase from controlled static tasks to dynamic, sport-relevant movements.
Saki et al. 2022 [36]	EG: 19.37 ± 0.32, CG: 20.32 ± 0.09	EG: 69.35 ± 5.42, CG: 66.41 ± 3.75	EG: 179.39 ± 5.36, CG: 178.42 ± 4.71	NR	N/R	The Sports-Metrics™ soccer protocol in this study lasts 6 weeks (18 sessions) with three sessions per week. It is organized into four main categories: agility/reaction drills (e.g., zig-zag and obstacle runs with coach commands), speed/endurance/resistance running (e.g., shuttle runs and running with resistance), ladder drills (progressing through different stepping patterns), and plyometrics plus resistance/strength work. Plyometric training progresses from two-foot jumps (including variations with rotation) to single-leg jumps, and the strength/resistance component increases in work duration from 30 s (sessions 1–6) to 45 s (7–12) and then 60 s (13–18) per exercise.
Mohammadi et al. 2022 [31]	EG: 20.08 ± 2.02, CG: 19.1 ± 2.71	EG: 56.17 ± 4.86, CG: 53.50 ± 5.35	EG: 164.1 ± 7.34, CG: 162.3 ± 5.35	EG: 20.88 ± 1.89, CG: 20.30 ± 1.81	N/R	6-week Sport-Metrics training program combined with strength exercises. The weekly program includes agility/reaction drills (e.g., zig-zag runs, runs between obstacles with coach commands), speed/acceleration and running drills (e.g., shuttle runs, backpedal runs, jogging/linear runs, and runs with resistance bands), ladder drills (e.g., varied ladder stepping patterns), and plyometric drills (progressing from two-foot jumps to single-leg jumps). The difficulty and intensity increase from week 1 to week 6. The strength component includes squats, lunges, calf raises, hamstring exercises (prone and supine), abdominal/core training, hip exercises with resistance bands, and wall sits, with exercise duration gradually increasing from 30 s in the early weeks to 60 s in the final weeks.
Shahrjerdi et al. 2021 [32]	EG: 15.92 ± 1.18, CG: 17.75 ± 2.63	EG: 57.15 ± 7.87, CG: 58.25 ± 7.41	EG: 167 ± 6.4, CG: 165 ± 4.8	EG: 20.37 ± 3.18, CG: 21.18 ± 2.74	Financial support from the Arak University	The Sports-metrics training protocol for female volleyball players is a 6-week program combining plyometric and agility drills with progressive intensity. Each week includes several sessions featuring sport-specific movements such as resisted partner pushes, fast and backward running, ladder and rotation drills, shuttle and reaction-based drills (e.g., boxing to coach commands), and jumping/footwork exercises. In parallel, a strength component is performed across the sessions using resistance-band and partner-resisted exercises (e.g., squats, lunges, single-leg calf raises, hamstring bridges, scapular work, core stability, hip flexor/abductor and boxing-footwork). Time per strength exercise progresses from 30 s in sessions 1–6 to 45 s in sessions 7–12, and then to 60 s in sessions 13–18.
Saki et al. 2021 [35]	EG: 18.40 ± 0.5, CG: 18.64 ± 1.08	EG: 70.47 ± 9.62, CG: 64.64 ± 6.61	EG: 180.40 ± 6.86, CG: 179.57 ± 6.72	EG: 21.59 ± 2.14, CG: 20.04 ± 1.73	N/R	The Sports-Metrics Soccer-Training (SMST) protocol is a progressive 18-session program that combines agility/reaction, endurance-speed-resistance, ladder drills, plyometrics, and strength exercises. In the first part of the program, agility and reaction drills progress from zigzag runs and obstacle running to more complex tasks such as Nebraska, Illinois, mirror press, and T-drill patterns, while endurance and speed work includes backward running, jogging, resisted running, short-step running, band-assisted acceleration, and shuttle-based drills with increasing complexity. The second part of the program continues with ladder drills that advance from simple up-up/back-back and toe-touch patterns to outside-foot, inside-outside, and one-foot forward/backward sequences. Plyometric training also progresses gradually from two-foot jumps to open two-foot jumps, rotational jumps, and single-leg jumps, and the strength component includes band-resisted squats and lunges, calf and hamstring strengthening, abdominal work, hip flexion/abduction, arm strengthening, and wall sits with duration increasing from 30 to 60 s across sessions.
Singh et al. 2015 [39]	20.0 ± 1.2	52.1 ± 4.9	163.4 ± 4.4	N/R	N/R	This protocol is a 6-week training program with 3 sessions per week, focused on plyometric jumps and agility work with a principle of progressive overload. Weeks 1–2 include fundamental jumps (wall jump, tuck jump, squat jump, barrier jumps, lateral/forward-backward hops, 180° jumps, broad jumps, and bounding), with Week 2 adding 5 s to each jump and 5 extra repetitions to broad jumps. Weeks 3–4 introduce more advanced movements such as triple broad-to-vertical jumps, single-leg hops, scissor jumps, and distance bounding; Week 4 further increases the duration of all jumps and adds 3 repetitions to the triple broad-to-vertical jump. Weeks 5–6 include step-jump up/down/vertical work, mattress jumps, triple single-leg hop with stick, and multiple runs of bounding, with Week 6 adding 5 repetitions to the step-jump sequence and 1 extra bounding run. Across all weeks, various agility drills (shuttle/maze, tip/figure-4, square/4-dot, defensive slides/shoot-and-sprint, Iris D, and T-drill) support improvements in explosive power, neuromuscular control, and overall agility.

Six studies exclusively enrolled female athletes [31–34, 38, 39] and three studies included male athletes [35–37]. The intervention duration was consistent across all studies, consisting of 6 weeks with three sessions per week. This methodological consistency may reduce heterogeneity related to intervention exposure and improve comparability across studies. However, this interpretation should be considered cautiously, as similarities in study design and intervention delivery among studies originating from the same or closely related research groups may also have contributed to the observed reduction in heterogeneity.

Among the nine included studies, the type of athletes was reported inconsistently. Specifically, three studies included soccer players [35, 36, 38], three studies involved volleyball players [31–33], two studies examined basketball players [34, 39], and one study involved futsal players [37]. The variability and inconsistent reporting of athlete type limited the ability to perform subgroup analyses based on sport discipline. This heterogeneity in participant characteristics may also affect the external validity of the pooled estimates, as the results likely reflect a mixed athletic population and may not be directly generalizable to specific sport disciplines.

### Methodological quality assessment

The two randomized controlled trials evaluated using the PEDro scale demonstrated good methodological quality, with both studies scoring 7 out of 10 on the PEDro scale. The primary methodological limitations were related to concealed allocation and the absence of participant and therapist blinding. The quasi-experimental studies demonstrated low to moderate methodological quality based on the JBI checklist (scores: 4–6/9), primarily due to limitations in confounding control, blinding, and incomplete reporting of intervention and statistical procedures. Detailed risk-of-bias assessments are provided in Supplementary File 2.

### Risk of bias

The risk of bias assessment for the included studies is presented in Table 2. RCTs were evaluated using the RoB 2. Both included RCTs (Saki et al., [35]; Saki et al., [36]) demonstrated an overall judgment of “some concerns” for risk of bias. Most domains were judged as low risk of bias; however, at least one domain in each study raised some concerns, primarily related to methodological limitations in the study design and/or outcome assessment procedures. These methodological concerns should be considered when interpreting the pooled findings, as they may reduce confidence in the precision and robustness of the estimated intervention effects.

**Table 2:** Risk of bias for included studies using the RoB-2 for RCTs

Authors/RoB-2 (RCT)	Q1	Q2	Q3	Q4	Q5	*Overall*
Saki et al. 2022 [36]	L	S	L	L	L	Some concern
Saki et al. 2021 [35]	L	S	L	L	L	Some concern

*Abbreviations*: *L* low, *NA* Not applicable, *S* Some

Non-randomized studies were assessed using ROBINS-I in Table 3. Most studies demonstrated concerns related to confounding and participant selection, resulting in an overall judgment of serious risk of bias across the included quasi-experimental studies. These methodological limitations should be considered when interpreting the pooled findings, as a serious risk of bias may reduce the certainty of the evidence, increase susceptibility to residual confounding, and potentially influence the magnitude and stability of pooled effect estimates.

**Table 3:** Risk of bias for included studies using the ROBINS-I for non-RCTs

Authors	Q1	Q2	Q3	Q4	Q5	Q6	Q7	*Overall*
Kalantariyan et al. 2025 [37]	L	S	L	L	NI	L	L	Serious risk
Mohammadi et al. 2024 (a) [33]	L	S	L	L	NI	L	L	Serious risk
Mohammadi et al. 2024 (b) [38]	L	S	L	L	NI	L	L	Serious risk
Mohammadi et al. 2023 [34]	L	S	L	L	NI	L	L	Serious risk
Mohammadi et al. 2022 [31]	L	S	L	L	NI	L	L	Serious risk
Shahrjerdi et al. 2021 [32]	L	S	L	L	NI	L	L	Serious risk
Singh et al. 2015 [39]	L	S	L	L	NI	L	L	Serious risk

*Abbreviations*: *L* Low, *S* Serious, *NI* No information

### GRADE assessment

According to the GRADE framework, the overall certainty of evidence was low. Low-certainty evidence supported changes in DKV, LESS, agility, dynamic balance, power, and speed (due to generally consistent findings and low heterogeneity across most outcomes, with downgrading primarily related to potential publication bias and study design limitations); whereas, the certainty of evidence for core strength was very low (due to moderate heterogeneity and methodological limitations). The detailed GRADE assessments for each outcome are provided in Supplementary File 3.

### Heterogeneity, publication bias, and sensitivity analysis

No substantial heterogeneity was observed for DKV, LESS, agility, dynamic balance, power, or speed outcomes (I² = 0%). However, despite methodological and clinical variability across studies, these findings should be interpreted cautiously. The low observed heterogeneity may partly reflect similarities in intervention structure, outcome direction, study design, or overlap in methodological approaches across studies, rather than true homogeneity of effects. In contrast, moderate heterogeneity was identified for core strength outcomes (I² = 64%). Differences in core strength assessment methods, testing protocols, exercise implementation, and participant characteristics across studies may have contributed to the observed variability in pooled estimates.

Publication bias was explored using Egger’s and Begg’s tests. However, it should be emphasized that all pooled analyses included fewer than ten studies, and under these circumstances, publication bias tests have very limited statistical power and may produce unstable results. Therefore, the findings of these tests should be interpreted with caution and should not be overinterpreted. With this important limitation in mind, Egger’s test suggested potential publication bias for LESS and dynamic balance outcomes (*p* < 0.05), whereas Begg’s test did not reveal significant publication bias for these variables. For the analysis of agility outcomes, Begg’s test suggested potential publication bias, whereas Egger’s test was not statistically significant. No evidence of publication bias was observed for the remaining outcomes.

Sensitivity analyses demonstrated that the pooled results remained stable after sequential removal of individual studies. Sensitivity analyses indicated that the pooled estimates for DKV, LESS, agility, dynamic balance, power, and speed remained statistically significant after sequential removal of individual studies. Core strength remained non-significant. The detailed sensitivity analyses are provided in Supplementary File 4. Additional sensitivity analyses excluding serious-risk quasi-experimental studies were also performed, and the overall direction and significance of the pooled estimates remained largely unchanged.

### Outcome measures

#### Synthesized outcomes

Among the nine [31–39] included studies, three evaluated DKV [31, 33, 38], three assessed LESS outcomes [31–33], three assessed core strength [34, 36, 38], four investigated agility performance [34, 35, 37, 39], three measured dynamic balance [34, 37, 38], three evaluated power [34, 35, 37], and two assessed speed-related performance outcomes [34, 35]. Due to variations in outcome measures and testing protocols across studies, separate meta-analyses were conducted for each outcome domain. The number of studies included in each pooled analysis varied according to the availability of comparable data reported in the original articles.

#### Lower-limb kinematics outcomes

**Fig. 2:**
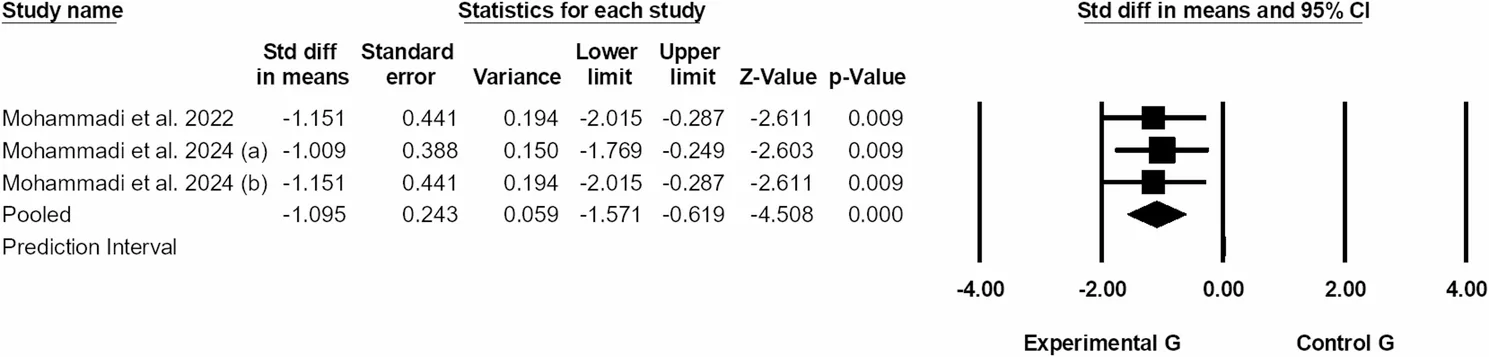
Forest plot regarding the comparison of the experimental group and the control group in DKV

**Fig. 3:**
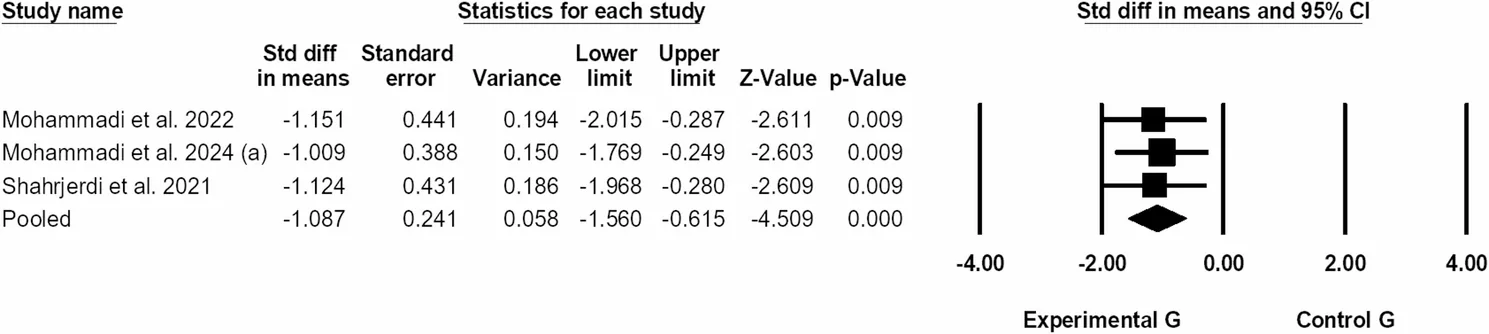
Forest plot regarding the comparison of the experimental group and the control group in LESS

Pooled analysis reflected changes in surrogate biomechanical outcomes, DKV (SMD: -1.09, 95% CI: -1.57 to -0.61, *p* < 0.0001) (Fig. 2) [31, 33, 38] and LESS (SMD: -1.08, 95% CI: -1.56 to -0.61, *p* < 0.0001) (Fig. 3) [31–33].

#### Functional performance outcomes

**Fig. 4:**
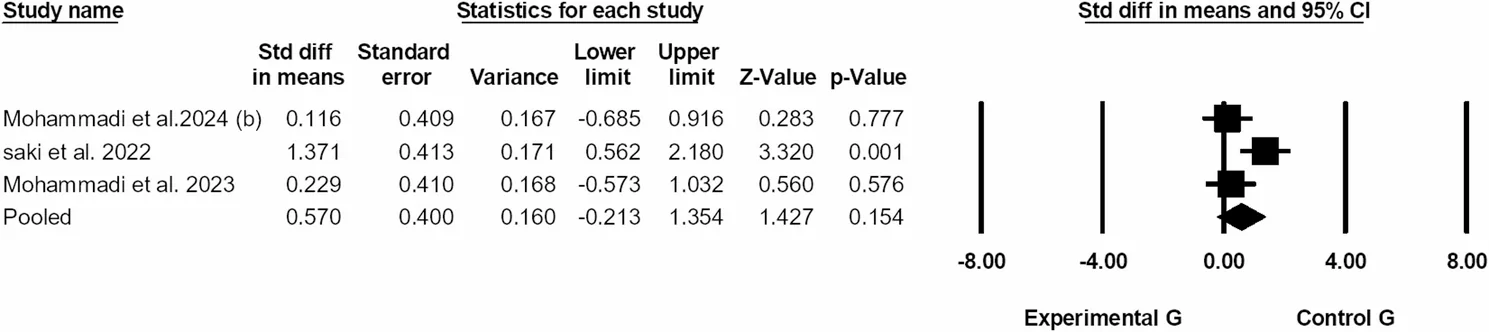
Forest plot regarding the comparison of the experimental group and the control group in core strength

**Fig. 5:**
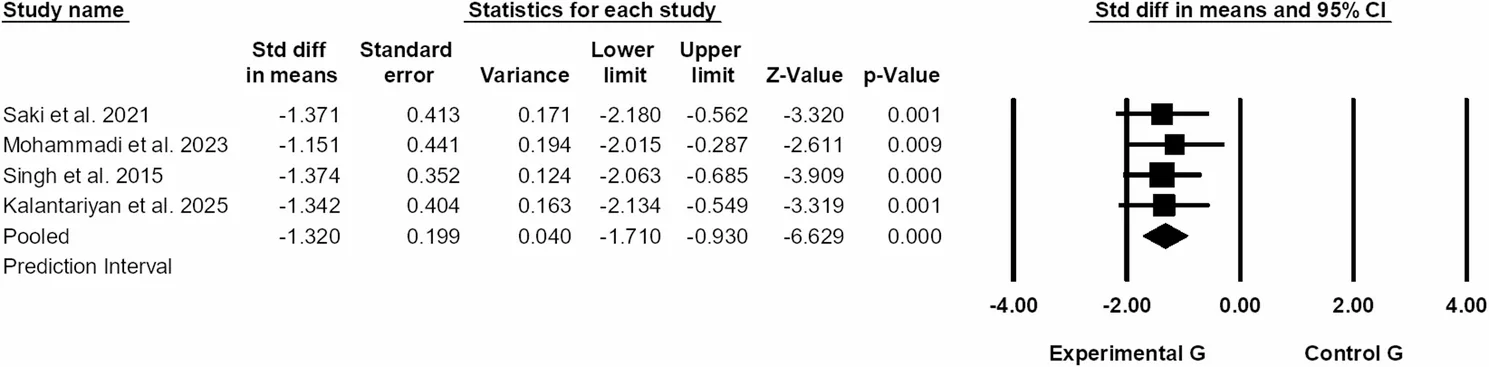
Forest plot regarding the comparison of the experimental group and the control group in agility

The pooled analysis demonstrated no changes in core strength (SMD: 0.57, 95% CI: -0.21 to 1.35, *p* = 0.154) (Fig. 4) [34, 36, 38]. Moreover, this finding should be interpreted cautiously, given the presence of moderate to high heterogeneity and the very low-certainty of evidence reported for this outcome, which may limit the robustness and generalizability of the pooled estimate. In addition, agility performance significantly changed (SMD: -1.32, 95% CI: -1.71 to -0.93, *p* < 0.0001) (Fig. 5) [34, 35, 37, 39]. It is worth noting that different studies employed varying agility assessment tools, which may introduce construct heterogeneity and limit the comparability of pooled estimates across studies.

**Fig. 6:**
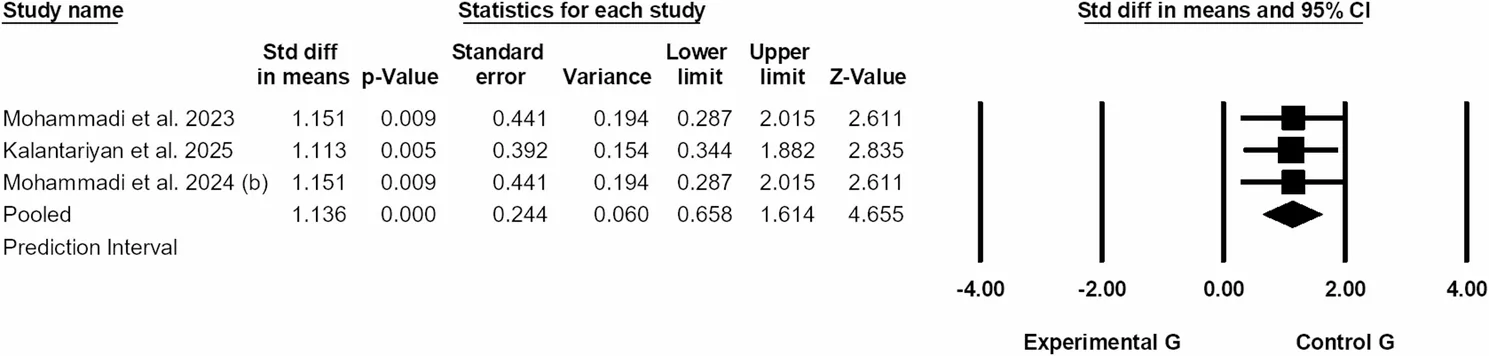
Forest plot regarding the comparison of the experimental group and the control group in dynamic balance

**Fig. 7:**
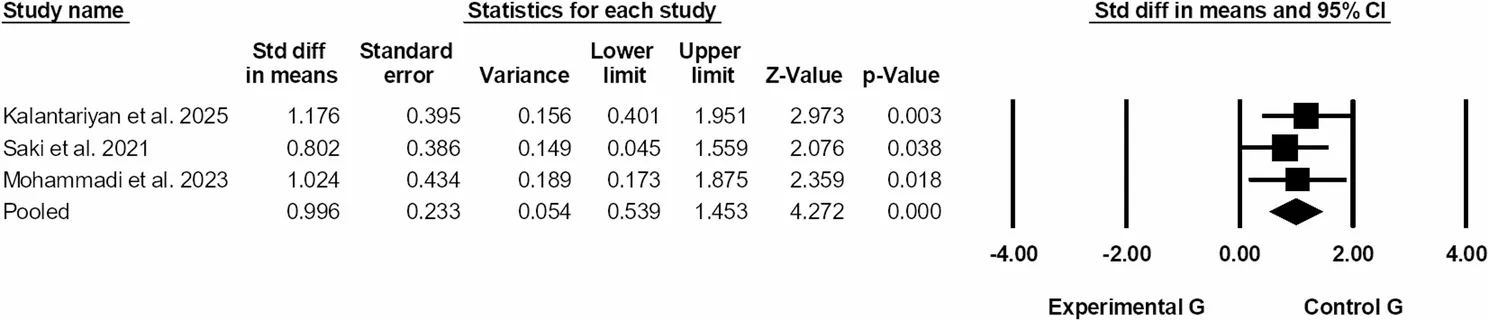
Forest plot regarding the comparison of the experimental group and the control group in power

**Fig. 8:**
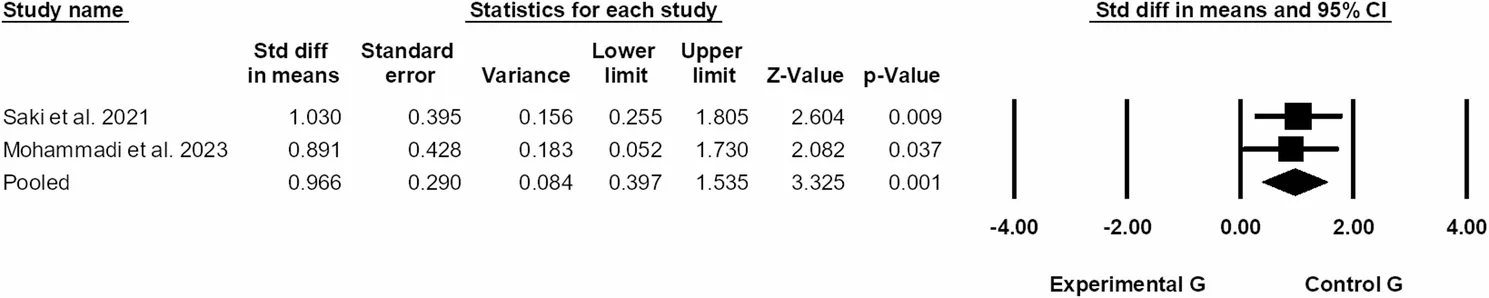
Forest plot regarding the comparison of the experimental group and the control group in speed

Furthermore, the meta-analysis demonstrated a significant increase in dynamic balance (SMD: 1.13, 95% CI: 0.65 to 1.61, *p* < 0.0001) (Fig. 6) [34, 37, 38]. It should be noted that different studies employed various clinical and field-based assessments of dynamic balance, which are generally considered proxy measures of dynamic postural control. While these tests assess related constructs, some degree of variability in the specific aspects of balance measured across studies may have influenced construct consistency within the pooled estimate. Significant changes were also observed in power (SMD: 0.99, 95% CI: 0.53 to 1.45, *p* < 0.0001) (Fig. 7) [34, 35, 37] and speed performance (SMD: 0.99, 95% CI: 0.39 to 1.53, *p* = 0.001) (Fig. 8) [34, 35]. It should be acknowledged that different studies used varying assessment methods to evaluate power (e.g., vertical jump and Sargent jump) and speed (e.g., sprint tests over different distances and sport-specific performance tests). These methodological differences may have introduced construct variability and could have influenced the magnitude and comparability of the pooled effect estimates.

**Fig. 9:**
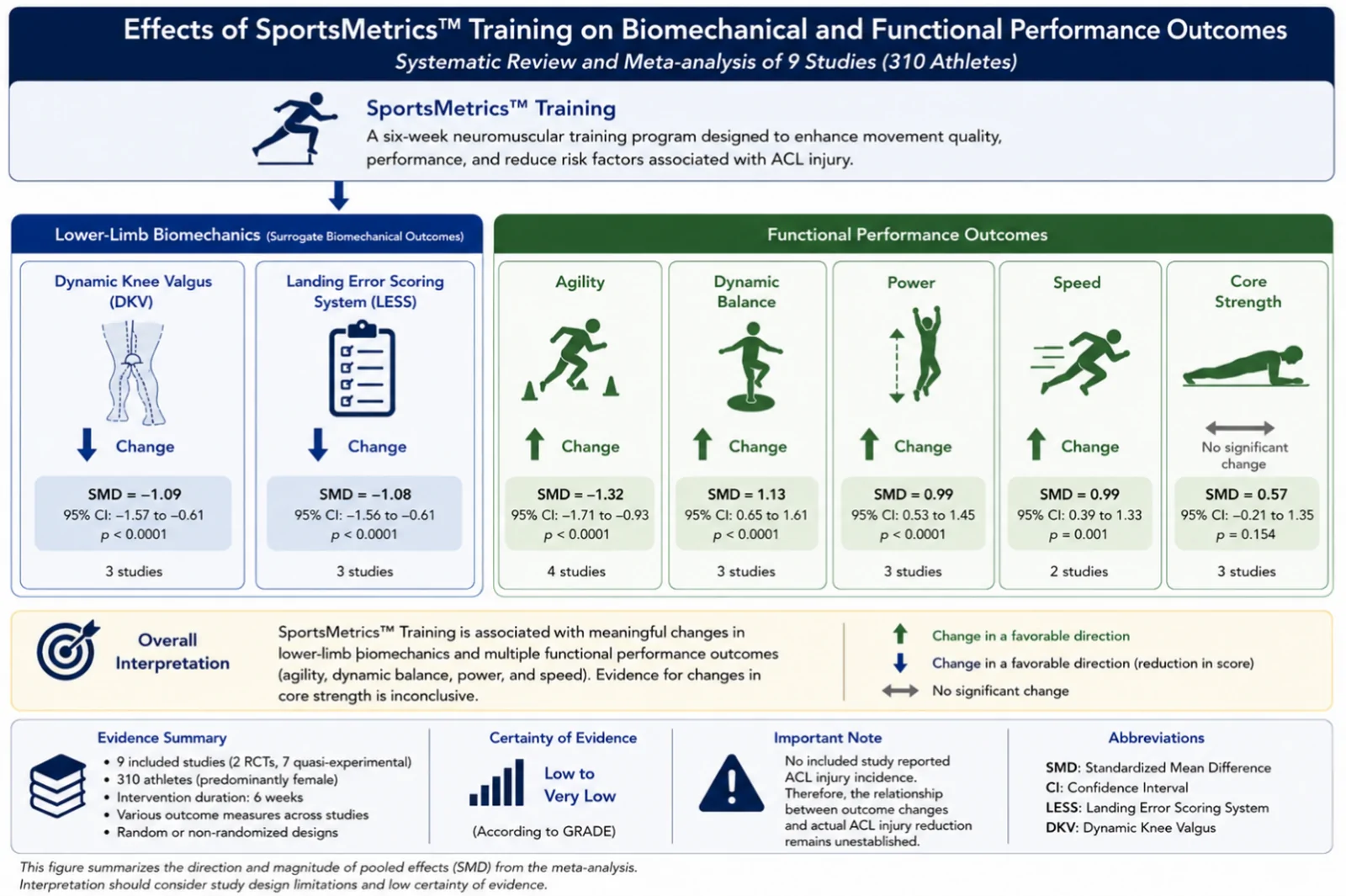
Graphical summary of the pooled effects of SMT on lower-limb biomechanics and functional performance outcomes

To facilitate the interpretation and clinical applicability of the findings, a graphical summary of the pooled effects across all outcome domains is presented in Fig. 9. This figure provides an overview of the direction and magnitude of the observed changes following SMT based on the pooled meta-analytic results.

## Discussion

The present systematic review and meta-analysis is the first to quantitatively synthesize the effects of SMT on lower-extremity kinematics and functional performance outcomes in athletes. The findings associated with improvement in high-risk landing mechanics (DKV, LESS) and agility, balance, power, and speed, whereas core strength remained non-significant. However, these findings should be interpreted within the context of methodological limitations, including the predominance of non-randomized studies and limited sample sizes.

### Lower-limb kinematics outcomes

Clinically important findings were observed for DKV and LESS, both of which demonstrated significant changes. These findings are clinically important because excessive DKV and poor landing mechanics are recognized as major biomechanical contributors to non-contact ACL injury mechanisms, particularly in female athletes during jump-landing and cutting tasks [40]. SMT can be related to these variables through a combination of plyometric training, landing retraining, strength exercises, agility drills, and neuromuscular control tasks. Compared with commonly used neuromuscular warm-up programs, SMT repeatedly exposes athletes to high-demand landing and deceleration situations, which may be associated with improvements in movement patterns and sport-specific performance outcomes [35]. These changes are often interpreted within a motor learning framework; however, motor learning processes were not directly assessed in the included studies.

The reduction in DKV observed in this review is consistent with previous neuromuscular training literature reporting changes in frontal-plane knee control following plyometric and injury-prevention interventions [41]. However, the magnitude of changes appears larger than that commonly reported for programs such as FIFA 11+ (male *p* = 0.28, female *p* = 0.001) [42], or generalized neuromuscular warm-up interventions [43, 44]. One possible explanation is that SMT integrates progressive plyometric loading, unilateral landing drills, rapid deceleration tasks, and multi-directional movement patterns that may more directly target movement deficits linked to high-risk landing mechanics [45]. In addition, several included studies recruited athletes with pre-existing valgus deficits, which may partly explain the greater changes compared with studies involving healthy asymptomatic athletes.

The observed improvements may be partially explained by several neuromuscular and neurophysiological adaptations induced by integrative neuromuscular training. Repeated exposure to plyometric, balance, and landing-control tasks may be associated with improvements in movement patterns and functional performance outcomes [46]. Additionally, proprioceptive adaptations may contribute to enhanced postural control and movement accuracy [47]. Repetitive stretch-shortening cycle loading during plyometric activities may also improve musculotendinous stiffness and elastic energy utilization, thereby enhancing explosive and functional performance outcomes [48]. Furthermore, improvements in lower-extremity control may be associated with enhanced activation patterns of key stabilizing musculature, particularly the gluteal and hamstring muscle groups [49, 50]. However, these mechanisms remain hypothesis-generating because the included studies did not directly assess neurophysiological processes, such as sensorimotor integration or muscle activation patterns.

Similarly, the significant change in LESS suggests that SMT has a positive influence on overall landing quality and movement efficiency. LESS is a valid and reliable clinical assessment tool of jump-landing biomechanics [12]. Therefore, improvements in LESS likely indicate broader neuromuscular adaptations beyond isolated frontal-plane knee control [51]. The repeated exposure to jump-landing retraining and feedback-oriented plyometric exercises within SMT may enhance movement coordination, proprioception, and lower-extremity alignment during high-impact tasks.

The neuromuscular adaptations following SMT may be further contextualized by recent evidence on motor control recovery after ACL injury. Wei et al. [52] demonstrated that neuromuscular coordination following ACL reconstruction follows a staged recovery process, from early compensatory quadriceps-dominant patterns to gradual normalization by 12 months post-surgery. This underscores the potential value of SMT in facilitating the normalization of motor module organization, although this was not directly assessed in the included studies.

### Functional performance outcomes

SMT was associated with changes in several functional performance outcomes, including agility, dynamic balance, power, and speed. However, the observed changes in core strength require cautious interpretation, given the moderate heterogeneity and methodological limitations across studies. This finding may be explained by the frequent incorporation of trunk stabilization exercises, single-leg control tasks, perturbation-based activities, and dynamic postural challenges within SMT protocols [53]. Alterations in core strength are hypothesized to be associated with improved proximal stability and movement control [54]. However, these mechanisms were not directly assessed in the included studies.

Agility also demonstrated a significant change. These findings may be explained by the multidirectional movement demands, rapid deceleration tasks, and change-of-direction drills included in SMT protocols, which are closely related to agility performance [55]. However, these findings may be influenced by variations in agility assessment protocols across studies, including the Illinois test, T-test, and sport-specific change-of-direction tasks. In addition, agility performance depends not only on neuromuscular qualities but also on perceptual and cognitive decision-making abilities, including visual scanning, anticipation, and response selection, which may not fully adapt during short-term interventions [56]. Moreover, the agility tests used in the included studies (e.g., Illinois test, T-test) are predominantly pre-planned change-of-direction tasks that do not adequately capture reactive agility or decision-making demands inherent in actual sport situations [57, 58]. This distinction is clinically relevant, as reactive agility, rather than pre-planned agility, may be more closely associated with injury-prone situations. The absence of reactive agility assessments in the included studies limits the ecological validity of the observed findings.

The changes in dynamic balance may be explained by enhanced trunk control, proprioceptive function, and lower-extremity stability developed through single-leg tasks, perturbation-based drills, and multi-planar movement exercises included in the program [59, 60]. Balance changes may be considered functionally relevant, as impaired postural control has been associated with movement patterns that are linked to altered lower-extremity mechanics during landing and cutting tasks [61]. These findings may therefore represent improvements in movement control rather than confirmed reductions in injury risk.

The significant changes in power and speed may be explained by the strong emphasis on plyometric and explosive-training components within SMT protocols. Repeated stretch-shortening cycle activities are commonly hypothesized to contribute to improvements in neuromuscular function and performance-related adaptations [62]. However, these mechanisms cannot be confirmed because they were not directly assessed in the included studies. Consequently, observed improvements in sprinting and jumping performance should be interpreted at the level of functional outcomes rather than confirmed physiological adaptations.

### Heterogeneity, publication bias, and certainty of evidence

The magnitude of pooled effects, particularly for DKV and LESS, warrants careful interpretation. The large effect sizes observed in this meta-analysis, SMD of -1.09 for DKV and − 1.08 for LESS, should be interpreted with particular caution. Effect sizes of this magnitude are unusual in the neuromuscular training literature and are consistent with the well-documented phenomenon of effect size inflation in small, non-randomized studies. Several factors may contribute to this overestimation: (a) the predominance of quasi-experimental designs lacking randomization, which are susceptible to selection bias and regression to the mean; (b) relatively small sample sizes in most included studies, which are known to produce unstable and inflated effect estimates; and (c) the inclusion of athletes with pre-existing biomechanical deficits (e.g., baseline DKV ≥ 8°), which may exaggerate pre-to-post changes compared to studies involving healthy asymptomatic populations.

Heterogeneity was generally low across most pooled outcomes, with I² = 0% observed for DKV, LESS, agility, dynamic balance, power, and speed. This finding requires careful interpretation, as it is uncommon despite apparent clinical and methodological diversity across studies. Several factors may explain this pattern. First, genuine methodological similarities may have contributed, as most studies adopted comparable SMT protocols (2–3 sessions/week, 6–8 weeks duration, similar exercise selection) and used standardized outcome assessments (2D motion analysis for kinematics, validated field tests for functional performance). Second, the predominance of small, quasi-experimental studies may artificially suppress heterogeneity estimates, as sampling error and within-study variance are larger in small samples, leading to unstable I² values. Third, studies originating from similar research environments may introduce clustering effects not captured by conventional heterogeneity statistics.

Moreover, I² = 0% should not be misinterpreted as evidence of true homogeneity. Clinical diversity was evident across studies, including differences in sport type, participant sex, and baseline characteristics (e.g., inclusion of athletes with DKV ≥ 8° versus asymptomatic athletes). However, I² values are influenced by sample size, study design, and measurement precision, and may therefore underestimate true clinical heterogeneity.

Publication bias was explored using Begg’s and Egger’s tests; however, the interpretation of these findings should be approached with great caution because all pooled analyses included fewer than ten studies. Under these circumstances, publication bias tests have very limited statistical power and may produce unstable and unreliable results. Therefore, no meaningful conclusions regarding the presence or absence of publication bias can be drawn from these analyses, and these results should be considered preliminary.

According to the GRADE framework, the certainty of evidence ranged from very low to low across outcomes. Low-certainty evidence supported improvements in DKV, LESS, agility, dynamic balance, power, and speed. Nevertheless, sensitivity analyses demonstrated relatively stable pooled estimates across most outcomes. However, this stability should be considered alongside the limited number of included studies and the predominance of studies with a serious risk of bias according to ROBINS-I. Therefore, consistency of pooled estimates should not be interpreted as certainty of evidence.

### Clinical implications

From a practical perspective, the findings suggest that SMT may be considered by coaches, physiotherapists, and strength and conditioning specialists as a training approach that can influence movement quality and athletic performance-related outcomes. This combination of performance-related and injury-risk-related surrogate outcomes could be relevant for adherence, given that athletes sometimes avoid prevention programs perceived to impair performance. Unlike some traditional injury-prevention models that primarily focus on the modification of injury-related biomechanical deficits [63], SMT appears to integrate performance enhancement and neuromuscular training within the same framework.

The findings may be particularly relevant for athletes demonstrating biomechanical deficits such as DKV or poor landing control. Given that most included studies involved soccer, volleyball, basketball, and futsal athletes, SMT may be particularly applicable in sports characterized by repetitive jump-landing and cutting maneuvers. However, this interpretation reflects potential applicability based on the included populations rather than the demonstrated superiority of SMT within specific sports contexts.

It is also important to acknowledge that the present review compared SMT exclusively against control conditions (usual training or no intervention). Therefore, the current evidence does not allow any conclusions regarding the relative effectiveness of SMT compared with other established injury-prevention programs, such as FIFA 11+, PEP, HarmoKnee, or Knäkontroll. From a clinical perspective, practitioners are often faced with choosing between multiple available programs, and head-to-head comparisons are needed to inform such decisions. Until such comparative studies are conducted, the findings of this review should be interpreted as evidence that SMT may be a viable option, but not necessarily a superior one.

It should be considered that the included studies involved athletes from soccer, volleyball, basketball, and futsal sports with different movement patterns and ACL injury mechanisms. Soccer and futsal involve frequent cutting, pivoting, and deceleration [64]; volleyball is characterized by repetitive jump-landing tasks [65]; and basketball combines multidirectional movements with high-impact landings [66]. Despite these differences, consistent improvements were observed across most outcomes, suggesting broad applicability of SMT. However, sport-specific effects could not be directly examined due to the limited number of studies per sport.

### Relationship between surrogate outcomes and actual ACL injury risk

In the present meta-analysis, significant improvements were observed in surrogate outcomes such as reduced DKV and LESS scores. These variables are widely recognized in the literature as biomechanical indicators associated with non-contact ACL injury mechanisms [6, 7]. Furthermore, it is important to recognize that the biomechanical assessments employed in the included studies, such as standardized drop-jump tasks, double-leg squats, and LESS scoring, are primarily closed, predictable, and self-paced movements. These assessments do not adequately replicate the high-speed, reactive, and unpredictable environments in which most non-contact ACL injuries occur. During actual sport participation, athletes must simultaneously process visual information, make rapid decisions, and execute motor responses under time pressure, often while fatigued. This cognitive-motor coupling imposes dual-task demands that can compromise movement quality and increase injury risk [67], yet none of the included studies incorporated such ecologically valid assessments. Executive function, reactive agility, and decision-making speed under fatigue are increasingly recognized as independent contributors to ACL injury risk that are not captured by closed assessment protocols.

Prospective cohort studies have shown that DKV is associated with an increased risk of ACL injury in female athletes and may be considered one of several biomechanical factors contributing to injury risk [68]. However, ACL injury occurrence is multifactorial in nature and is unlikely to be fully explained by isolated biomechanical variables such as DKV alone. Recent advances in computational biomechanics have introduced data-driven frameworks for dynamic ACL injury risk assessment under real-world conditions. Xu et al. [69] developed a multimodal deep learning approach combining musculoskeletal modeling and wearable sensor data to predict ligament fatigue failure in real time, overcoming the limitations of static biomechanical assessments used in the included studies. This highlights the need for future research to incorporate ecologically valid, individualized assessment tools. Moreover, none of the studies included in this review reported actual ACL injury rates during follow-up. Overall, while it has been hypothesized that correcting these biomechanical deficits may be associated with a reduction in injury risk, this hypothesis remains unconfirmed due to the absence of prospective injury-registration data in the included studies. Accordingly, the current evidence does not allow definitive conclusions regarding ACL injury reduction, and adequately powered RCTs with long-term follow-up and injury registration are required before definitive injury-prevention recommendations can be made.

### Limitations of the study and future directions

Several limitations should be acknowledged. First, the number of included studies was relatively small, particularly for speed and LESS outcomes. Second, most studies used quasi-experimental designs, while only two RCTs were included. Third, the methodological quality of the included quasi-experimental studies was generally low to moderate, and most non-randomized studies demonstrated a serious risk of bias. Fourth, most participants were young female athletes with DKV, which limits the external validity of the findings. This population specificity should be considered when interpreting the results, as the observed effects may not be directly generalizable to male athletes, athletes without biomechanical deficits, recreational populations, or other sporting disciplines. Therefore, the applicability of SMT beyond similar high-risk female athletic populations remains uncertain. Fifth, biomechanical assessments were frequently based on 2D video analysis rather than three-dimensional motion capture systems. While 2D analysis is practical for field-based screening, it is susceptible to measurement error due to out-of-plane movements, projection angles, and marker placement inaccuracies, which may reduce the precision of kinematic measurements. Additionally, power and speed outcomes were pooled across different assessment methods. These tests represent related but not identical constructs; therefore, the pooled estimates should be interpreted as reflecting broader aspects of explosive performance and locomotor capacity rather than a single standardized measure. Sixth, all interventions lasted only six weeks, and none evaluated the long-term retention of biomechanical adaptations or subsequent injury incidence. It remains unclear whether the reported improvements persist over time or translate into long-term functional or injury-related outcomes.

Seventh, the evidence is very low to low certainty, mostly derived from non-randomized studies with a serious risk of bias. Eighth, no included study reported actual ACL injury incidence outcomes, which represents a major limitation of the current evidence base. Furthermore, the observed I² = 0% across most outcomes should be interpreted cautiously, as this may reflect small-study effects or methodological artifacts rather than true homogeneity, particularly given the limited number of included studies and predominance of non-randomized designs. Additionally, the limited number of studies per sport precluded subgroup analyses. The influence of sport-specific characteristics, such as movement patterns, landing mechanics, and injury mechanisms, on treatment response could not be assessed. Accordingly, the generalizability of the findings to individual sports remains uncertain. Finally, although participant overlap was considered unlikely, studies from the same research groups may introduce potential clustering effects that could influence the generalizability of the pooled findings.

Future studies should prioritize high-quality randomized controlled trials with larger sample sizes and longer follow-up periods. Direct head-to-head comparisons between SMT and other established neuromuscular training programs, such as FIFA 11+, PEP, HarmoKnee, and Knäkontroll, are also needed to determine their relative effectiveness and help clinicians make evidence-based decisions. In addition, future research should investigate sport-specific adaptations, sex-related responses, and the long-term transfer of biomechanical improvements to real-world injury reduction. The integration of 3D motion analysis, force-plate assessment, and kinetic variables may further clarify the mechanisms underlying SMT-induced adaptations.

### Conclusion

SMT appears to be associated with improvements in landing biomechanics (DKV and LESS) and several functional performance outcomes, including agility, dynamic balance, power, and speed, particularly in athletes with baseline DKV deficits. However, core strength did not show significant changes. Nevertheless, the certainty of evidence remains low to very low because of methodological limitations and the absence of prospective injury outcomes. Therefore, current evidence is insufficient to support widespread implementation of SMT as a standard ACL injury-prevention strategy. High-quality randomized controlled trials with long-term follow-up and injury-related outcomes are warranted.

## Supplementary Information

Supplementary Material 1: Supplementary File 1. Overlap potential.

Supplementary Material 2: Supplementary File 2. The PEDro score.

Supplementary Material 3: Supplementary File 3. The GRADE.

Supplementary Material 4: Supplementary File 4. Sensitivity analysis.

Supplementary Material 5: Supplementary File 5. PRISMA 2020 checklist

## Data Availability

Detailed search results are available on request (Jalilimostafa3@gmail.com).

## Abbreviations

SMT: SportsMetrics training
ACL: Anterior Cruciate Ligament
ACLR: Anterior Cruciate Ligament Reconstruction
DKV: Dynamic Knee Valgus
LESS: Landing Error Scoring System
MJB: Mostafa Jalili Bafrouei
FZMG: Fatemeh Zahra Maleki Gohar
SHM: Seyed Hamed Mousavi
MR: Mohamad Rostami
FF: Florian Forelli
