# Supplementary material for: Effects of sportsmetrics training on biomechanical and functional performance outcomes related to ACL injury risk: a systematic review and meta-analysis
Source: BMC Sports Sci Med Rehabil. 2026 Aug 8;18:563. doi: 10.1186/s13102-026-01948-7 (PMC13737763; doi:10.1186/s13102-026-01948-7)
Supplement: Supplementary file 1 [file 13102_2026_1948_MOESM1_ESM.docx]

**Supplementary File 1. Assessment of potential participant overlap among studies from the same or closely related research groups**

To further evaluate the possibility of participant overlap among studies originating from the same or closely related research groups, we performed a detailed comparison of recruitment periods, recruitment sources, geographical locations, institutions, ethics approval codes, sport disciplines, participant characteristics, and sample sizes. Although several studies were conducted by the same research groups, no clear evidence of duplicate participant populations was identified based on the reported study characteristics. Differences in recruitment periods, recruitment settings, institutional affiliations, ethics approval identifiers, and participant characteristics suggest that participant overlap was unlikely; however, it could not be definitively excluded because individual participant-level data were unavailable. The detailed assessment is presented in the following table.

| **Research group/study** | **Recruitment period** | **Location/recruitment source** | **Institution and ethics approval** | **Sport** | **Sex** | **Sample size** | **Main participant characteristics** | **Assessment of potential overlap** |
| --- | --- | --- | --- | --- | --- | --- | --- | --- |
| Mohammadi et al. 2024a | September–October 2022 | Isfahan Province Volleyball Board, Isfahan, Iran | Kurdistan  University (IR.UOK.REC.1400.036) | Volleyball | Female | 30 | Club volleyball players with DKV, no injury during the previous year, at least 3 years of continuous club volleyball participation, and regular training three times per week | Although this study and other Mohammadi et al. studies included female athletes with DKV, this study recruited female volleyball players from the Isfahan Province Volleyball Board during September–October 2022. The other Mohammadi studies involved different sports (soccer, basketball, or volleyball from another province), different recruitment settings, and non-overlapping recruitment periods. The separate recruitment source, geographical location, and eligibility criteria suggest that participant overlap appeared unlikely, although it could not be definitively excluded. |
| Mohammadi et al. 2024b | April–June 2022 | Kurdistan Province Soccer Board, Kurdistan, Iran | Kurdistan  University  (IR.UOK.REC.1400.030) | Soccer | Female | 24 | Club soccer players with DKV, no serious lower-limb injury during the previous year, no major knee impairment, and at least 3 years of regular club football participation | This study recruited female soccer players from the Kurdistan Province Soccer Board between April and June 2022. Although another Mohammadi study also recruited athletes from Kurdistan Province, it involved a different sport (basketball), a different participant population, and an earlier recruitment period. Differences in sport discipline, recruitment criteria, and participant characteristics suggest that participant overlap appeared unlikely, although it could not be definitively excluded. |
| Mohammadi et al. 2023 | September–November 2021 | Kurdistan Province Basketball Board, Kurdistan, Iran | Kurdistan  University (IR.UOK.REC.1400.030) | Basketball | Female | 30 | Basketball players with DKV, tuck jump screening score ≥2, no lower-limb injury during the previous 12 months, no history of ligament surgery, and at least 3 years of regular basketball training | This study involved female basketball players recruited from the Kurdistan Province Basketball Board during September–November 2021. Other Mohammadi studies included different sports (soccer or volleyball), different recruitment periods, and different eligibility criteria. Therefore, despite originating from the same research group, differences in sport type, recruitment source, and participant characteristics suggest that participant overlap appeared unlikely, although it could not be definitively excluded |
| Mohammadi et al. 2022 | July–August 2021 | Kermanshah Province Volleyball Board, Kermanshah, Iran | Kurdistan  University (IR.UOK.REC.1400.030) | Volleyball | Female | 24 | Club volleyball players with DKV, no serious lower-limb injury during the previous 12 months, no major knee injury, no knee impairment, no history of ligament surgery, and at least 3 years of club volleyball participation | This study recruited female volleyball players from the Kermanshah Province Volleyball Board during July–August 2021. Although Mohammadi et al. 2024a also included female volleyball players, the studies were conducted in different provinces (Kermanshah vs Isfahan), involved different recruitment organizations, and used non-overlapping recruitment periods. These differences, together with separate ethics approval records, suggest that the datasets were likely independent, although participant overlap could not be definitively excluded. |
| Saki et al. 2022 | April–July 2020 | Kurdistan Province Soccer Board, Kurdistan, Iran | Kurdistan  University (IR.UOK.REC.1399.011) | Soccer | Male | 24 | Soccer players with at least 3 years of club-level participation, no serious injury in the previous year, and regular attendance in training sessions | This study recruited male soccer players from the Kurdistan Province Soccer Board between April and July 2020. Although Saki et al. 2021 also investigated male soccer players, the studies were conducted in different provinces (Kurdistan vs Hamedan), involved different recruitment sources, and had distinct recruitment periods. The differences in geographical location, recruitment setting, and study timelines participant overlap appeared unlikely, although it could not be definitively excluded |
| Saki et al. 2021 | June–September 2019 | Hamedan Province Soccer Board, Hamedan, Iran | Bu-Ali Sina University (IR.BASU.REC.1398.018) | Soccer | Male | 29 | Soccer players with more than 3 years of club-level experience, no knee or lower-limb injury, and regular participation in structured training programmes | This study recruited male soccer players from the Hamedan Province Soccer Board between June and September 2019. Although another Saki et al. study included male soccer players, it was conducted in a different province (Kurdistan), with a different recruitment source and a later recruitment period. The independent recruitment setting and separate ethics approval information support that the included participants suggest that this represented an independent cohort; however, participant overlap could not be definitively excluded. |

Abbreviations: DKV, dynamic knee valgus; ACL, anterior cruciate ligament.
