# Supplementary material for: Effects of sportsmetrics training on biomechanical and functional performance outcomes related to ACL injury risk: a systematic review and meta-analysis
Source: BMC Sports Sci Med Rehabil. 2026 Aug 8;18:563. doi: 10.1186/s13102-026-01948-7 (PMC13737763; doi:10.1186/s13102-026-01948-7)
Supplement: Supplementary file 2 [file 13102_2026_1948_MOESM2_ESM.docx]

**Supplementary File 2. Quality Assessment of included studies using the PEDro scale for RCT and the JBI for Quasi-experimental studies**

| Authors/PEDro | Q2 | Q3 | Q4 | Q5 | Q6 | Q7 | Q8 | Q9 | Q10 | Q11 | Overall (10) |
| --- | --- | --- | --- | --- | --- | --- | --- | --- | --- | --- | --- |
| Saki et al. 2022 | Y | N | Y | N | N | Y | Y | Y | Y | Y | 7 |
| Saki et al. 2021 | Y | N | Y | N | N | Y | Y | Y | Y | Y | 7 |

| Overall (9) | Q9 | Q8 | Q7 | Q6 | Q5 | Q4 | Q3 | Q2 | Q1 | Authors/JBI |
| --- | --- | --- | --- | --- | --- | --- | --- | --- | --- | --- |
| 5 | Y | Y | N | Y | N | N | Y | N | Y | Kalantariyan et al. 2025 |
| 4 | Y | N | N | Y | N | N | Y | N | Y | Mohammadi et al. 2024 (a) |
| 4 | Y | N | N | Y | N | N | Y | N | Y | Mohammadi et al. 2024 (b) |
| 6 | Y | Y | N | Y | N | Y | Y | N | Y | Mohammadi et al. 2023 |
| 4 | Y | N | N | Y | N | N | Y | N | Y | Mohammadi et al. 2022 |
| 4 | Y | N | N | Y | N | N | Y | N | Y | Shahrjerdi et al. 2021 |
| 5 | Y | N | N | Y | Y | Y | N | N | Y | Singh et al. 2015 |

*Y = yes; N = no;
