# Supplementary material for: Effects of sportsmetrics training on biomechanical and functional performance outcomes related to ACL injury risk: a systematic review and meta-analysis
Source: BMC Sports Sci Med Rehabil. 2026 Aug 8;18:563. doi: 10.1186/s13102-026-01948-7 (PMC13737763; doi:10.1186/s13102-026-01948-7)
Supplement: Supplementary file 3 [file 13102_2026_1948_MOESM3_ESM.docx]

**Supplementary File 3. Details of GRADE assessment**

| **№ of studies** | **Certainty assessment** | | | | | | **Assessment** | |  |
| --- | --- | --- | --- | --- | --- | --- | --- | --- | --- |
|  | **Study design** | **Risk of bias** | **Inconsistency** | **Indirectness** | **Imprecision** | **Grade** | **Rate (95% CI)** | **Certainty** |  |
| DKV (follow-up: 6 weeks) | | | | | | | | | |
| 3 | quasi-experimental | serious | not serious | not serious | not serious | Down grade | (SMD: -1.09, -1.57 to -0.61, p < 0.001) | ⨁⨁◯◯ Low |  |
| LESS (follow-up: 6 weeks) | | | | | | | | | |
| 3 | quasi-experimental | serious | not serious | not serious | not serious | Down grade | (SMD: -1.08, -1.56 to -0.61, p < 0.001) | ⨁⨁◯◯ Low |  |
| Agility (follow-up: 6 weeks) | | | | | | | | | |
| 4 | randomised trials and quasi-experimental | serious | not serious | not serious | not serious | Down grade | (SMD: -1.32, -1.71 to -0.93, p < 0.001) | ⨁⨁◯◯ Low |  |
| Dynamic balance (follow-up: 6 weeks) | | | | | | | | | |
| 3 | quasi-experimental | serious | not serious | not serious | not serious | Down grade | (SMD: 1.13, 0.65 to 1.61, p < 0.001) | ⨁⨁◯◯ Low |  |
| Power (follow-up: 6 weeks) | | | | | | | | | |
| 3 | randomised trials and quasi-experimental | serious | not serious | not serious | not serious | Down grade | (SMD: 0.99, 0.53 to 1.45; p < 0.001) | ⨁⨁◯◯ Low |  |

| Speed (follow-up: 6 weeks) | | | | | | | | | |
| --- | --- | --- | --- | --- | --- | --- | --- | --- | --- |
| 2 | randomised trials and quasi-experimental | serious | not serious | not serious | not serious | Down grade | (SMD: 0.99, 0.39 to 1.53; p = 0.001) | ⨁⨁◯◯ Low |  |

| Core strength (follow-up: 6 weeks) | | | | | | | | | |
| --- | --- | --- | --- | --- | --- | --- | --- | --- | --- |
| 3 | randomised trials and quasi-experimental | serious | serious | not serious | not serious | Down grade | (SMD: 0.57, 0.09 to 1.64, p = 0.027) | ⨁◯◯◯ Very low |  |
