# Supplementary material for: Effects of sportsmetrics training on biomechanical and functional performance outcomes related to ACL injury risk: a systematic review and meta-analysis
Source: BMC Sports Sci Med Rehabil. 2026 Aug 8;18:563. doi: 10.1186/s13102-026-01948-7 (PMC13737763; doi:10.1186/s13102-026-01948-7)
Supplement: Supplementary file 4 [file 13102_2026_1948_MOESM4_ESM.docx]

**Supplementary File 4. Details of Sensitivity analysis**

Sensitivity analysis comparing the experimental group and the control group in DKV.

Sensitivity analysis comparing the experimental group and the control group in LESS.

Sensitivity analysis comparing the experimental group and the control group in agility.

Sensitivity analysis comparing the experimental group and the control group in dynamic balance.

Sensitivity analysis comparing the experimental group and the control group in power

Sensitivity analysis comparing the experimental group and the control group in core strength
